# Oral but Not Intravenous Glucose Acutely Decreases Circulating Interleukin-6 Concentrations in Overweight Individuals

**DOI:** 10.1371/journal.pone.0066395

**Published:** 2013-06-12

**Authors:** Patrick J. Manning, Wayne H. F. Sutherland, Sheila M. Williams, Sylvia A. de Jong, Gavin P. Hendry

**Affiliations:** 1 Department of Medicine, Dunedin School of Medicine, University of Otago, Dunedin, New Zealand; 2 Department of Preventive and Social Medicine, Dunedin School of Medicine, University of Otago, Dunedin, New Zealand; Sapienza, University, Italy

## Abstract

**Background:**

Plasma interleukin-6 (IL-6) concentrations decrease acutely 1 h after ingestion of a glucose load or mixed meals and this may be mediated by an anti-inflammatory effect of insulin. The aim of the present study was to compare the effect of higher versus lower insulin levels on plasma IL-6 concentrations following oral compared with intravenous glucose administration in overweight/obese subjects.

**Methods and Findings:**

Fifteen subjects (12 women and 3 men) with BMI >28 kg/m^2^ were given an oral glucose load (75g) followed a week later by an intravenous infusion of glucose aimed at matching plasma glucose concentrations during the oral glucose load. A week later, they drank a volume of water equivalent to the volume consumed with the oral glucose load. Plasma glucose, insulin, nonesterified fatty acids, and IL-6 concentrations and blood hematocrit were measured at 30 minute intervals for 2 h following each intervention. Plasma IL-6 decreased (13–20%) significantly (P = 0.009) at 30 min to 90 min following the oral glucose load and did not change significantly following the other two interventions. The incremental area under the curve for plasma IL-6 concentrations following oral intake of glucose was significantly lower compared with concentrations following intravenous glucose (P = 0.005) and water control (P = 0.02). Circulating insulin concentrations were significantly (P<0.001) and 2.8 fold higher following oral compared with intravenous glucose administration.

**Conclusions:**

These data show that plasma IL-6 concentrations did not decrease during isoglycemic, intravenous glucose administration suggesting that the markedly higher circulating insulin levels and/or gut-related factors may mediate the acute decrease in plasma IL-6 after oral glucose intake in overweight/obese subjects.

**Trial Registration:**

Australian New Zealand Clinical Trials Registry ACTRN12612000491864

## Introduction

Interleukin-6 (IL-6) is a marker of systemic inflammation that is synthesized in several tissues and regulates innate immunity and the acute phase response [1]. Fasting plasma IL-6 levels are abnormally high in obesity and insulin resistance [2], and predict the development of type 2 diabetes [3] and coronary heart disease [4]. Systemic IL-6 concentrations change acutely in the postprandial state with an initial decline followed by a later increase at approximately 3–4 hours after the meal. This initial reduction in plasma IL-6 occurs after an oral glucose load [5], a mixed meal [6] and a fatty meal [7]. It is seen in both lean and obese individuals [6], as well as in those with diet controlled type 2 diabetes [8] and premature myocardial infarction [7].

The concomitant increase in circulating insulin levels is thought to contribute to the early decrease in plasma IL-6 after an oral glucose load [5] and after meals. Insulin has anti-inflammatory properties [9] that can attenuate the pro-inflammatory effect of hyperglycemia [10], [11]. In type 2 diabetic patients, an acute increase in circulating insulin levels during a meal decreases plasma concentrations of IL-6 and other inflammatory cytokines [12]. Conversely, when insulin secretion is suppressed, acute hyperglycemia during intravenous glucose administration increases plasma IL-6 concentrations [13]. If hyperinsulinemia following an oral glucose load is an important contributor to the early decrease in plasma IL-6 concentrations then this decrease should be attenuated when circulating insulin levels are substantially lower at the same blood glucose concentrations. Circulating concentrations of insulin at isoglycemia are markedly lower during intravenous glucose administration that does not stimulate release of incretins from the gut, compared with levels following an oral glucose load. Incretins are secreted from the gut during oral intake of nutrients and stimulate insulin secretion from the pancreas. The aim of the present study was to compare the response of plasma IL-6 concentrations during an oral glucose tolerance test with the response during isoglycemic, intravenous glucose administration in overweight to obese subjects. Our findings showed that plasma IL-6 concentrations were acutely and temporarily decreased following an oral glucose load when circulating insulin concentrations were markedly higher than levels during 2 h isoglycemic, intravenous glucose administration that did not appreciably affect plasma IL-6 concentrations.

## Methods

### Participants

This study was approved by the Lower Southern Regional Ethics Committee. Written informed consent was obtained from each subject before inclusion into the study. Fifteen overweight to obese adult participants (BMI>28 kg/m^2^) were recruited by newspaper advertisement. The sample size was based on our previous study (6). Exclusion criteria included known diabetes, cigarette smoking, current treatment with anti-inflammatory (or other medications), and clinical or biochemical evidence of acute or chronic illness.

### Study design

After an overnight fast, participants attended the research centre in the early morning and had anthropometric measurements performed. They then underwent the following procedures in the order on separate occasions and separated by at least 1 week:

A: Oral glucose load.

Participants ingested 75 g of oral glucose in 300 ml of water (Carbotest 75).

B: Intravenous Glucose Infusion

An intravenous cannula was placed and an infusion of 10% dextrose was commenced at a rate calculated according to the participants weight and 30 minute plasma glucose level obtained during the oral glucose tolerance test. Venous glucose concentrations were measured every 15 minutes and the infusion rate adjusted to match as closely as possible the glucose concentrations achieved during the oral glucose tolerance test. The infusion was discontinued at 120 minutes.

C: Water intake; control.

Participants ingested 300 ml of water.

Blood samples were taken at 0, 30, 60, 90 and 120 minutes during each of these procedures. Plasma glucose, insulin, nonesterified fatty acids (NEFA), haematocrit and IL-6 were measured in these blood samples.

### Laboratory methods

Venous blood samples were taken from participants into EDTA containing tubes and a plain tube. Tubes were centrifuged at 1500 g for 15 minutes at 4°C and plasma and serum were harvested. Aliquots of serum and plasma were stored at −80°C. Plasma glucose was measured by routine automated methods in the laboratories of Dunedin Hospital. Plasma insulin was measured on a Hitachi 911 autoanalyser using commercial kits and calibrators (Roche Diagnostics, Manheim, Germany). Plasma IL-6 was measured in duplicate by high sensitivity enzyme-linked immunosorbent assay methods using a commercial kit (R&D Systems, Minneapolis, MN). The intraassay coefficient of variation was 7% for IL-6. Plasma NEFA were measured using a commercial; kit (Roche Diagnostics). Samples from the same individual were measured in the same assay to reduce interassay variation.

### Statistical analysis

Data were expressed as mean±SD unless stated otherwise. The trapezium method was used to calculate incremental area under the curve (iAUC) [14]. A mixed model with a random effect for participant was used to compare iAUCs of variables among the treatments. Student's t-test was used to compare the iAUC of variables with zero. Repeated measures ANOVA on log transformed values was used to test for changes in values with time and to estimate carry-over by comparing zero-time values before the interventions. Spearman's rank correlation was used to test for relationships between changes in plasma IL-6 and insulin concentrations during the oral and intravenous administration of glucose. A regression model with random effect for participant was used to examine both the between subject and within subject correlation between iAUC plasma IL-6 and iAUC plasma insulin concentrations using combined oral and intravenous data. Two-sided test significance was used and a P value of <0.05 was considered to be statistically significant. Analyses were performed using Stata Statistical Software Release 12. College Station, TX: Stata Corporation; 2011.

## Results

The baseline characteristics of the participants are shown in Table S1. On average, the participants were obese, with a mean BMI >30 kg/m2, and had higher baseline concentrations of plasma IL-6 compared with values (1.4±0.6 ng/L) in a group of 14 lean subjects (mean BMI 22.9±1.9) of comparable ages (53±10 years) determined in our laboratory.

Figure S1 shows plasma glucose, insulin and NEFA concentrations and blood haematocrit and their iAUCs in participants during oral and intravenous glucose loads and ingestion of water (control). Despite attempting to match the glucose levels during oral and intravenous glucose administration, plasma glucose concentrations were significantly higher as summarised by a significantly (P = 0.03) higher iAUC, during intravenous glucose administration (Fig. S1A).

Serum insulin concentrations as summarised by their iAUCs were significantly (P<0.001) and higher during oral compared with intravenous glucose administration (Fig. S1B). Plasma NEFA concentrations as summarised by their iAUCs, were not significantly different (P = 0.65) during oral and intravenous administration of glucose (Fig. S1C). Blood hematocrit iAUC did not differ significantly from zero with oral glucose (P = 0.65) or water administration (P = 0.81) (Fig. S1D). Following intravenous glucose there was a small (−3%) decline in hematocrit as indicated by a significant (P = 0.004) decrease in iAUC that was significantly different compared with the iAUCs following oral glucose (P = 0.02) and water (P = 0.02) intake.

Figure S2 shows plasma IL-6 concentrations and iAUC during the study. The iAUC of plasma IL-6 concentrations during oral glucose intake was significantly (p = 0.005) lower compared with the corresponding iAUC during intravenous administration of glucose and compared with control iAUC (P = 0.02). The iAUC of plasma IL-6 concentrations was significantly (P = 0.01) less than zero and plasma IL-6 concentrations were significantly (P = 0.009) lower than baseline at 30, 60 and 90 minutes after ingestion of the glucose load in repeated measures ANOVA on log-transformed data. The iAUCs of plasma IL-6 concentrations during intravenous administration of glucose (P = 0.34) and following ingestion of water control (P = 0.59) were not significantly different from zero indicating that plasma IL-6 concentrations did not change significantly during these interventions.

Zero-time values of plasma concentrations of IL-6 (P = 0.69), glucose (P = 0.85), insulin (P = 0.24), and NEFA (P = 0.70) were not significantly different among the interventions.

The decreases in plasma IL-6 concentrations at 30 minutes (r = −0.111, n = 15, P = 0.69), 60 (r = 0.300, n = 15, P = 0.28) and 90 minutes (r = 0.307, n = 15, P = 0.27) after ingestion of glucose were not correlated significantly with the concomitant increases in serum insulin concentrations. Also, plasma IL-6 iAUC and was not significantly correlated with plasma insulin iAUC (regression coefficient = −0.00033, P = 0.33; R^2^ within = 0.109; R^2^ between = 0.0068) and plasma glucose iAUC (regression coefficient = 0.248, P = 0.48; R^2^ within = 0.0391; R^2^ between = 0.0057) in mixed model random-effects regression analysis in combined iAUC data during oral and intravenous loads.

## Discussion

These data in overweight, non-diabetic individuals show that the early acute decrease in plasma IL-6 following ingestion of a glucose load did not occur when serum insulin levels were substantially lower during intravenous administration of glucose to give glucose concentrations that were similar to those during oral glucose intake at all but one time point.

The decrease in plasma IL-6 concentrations in this study early after ingestion of glucose is in keeping with previous studies from this laboratory that have reported a decrease in plasma IL-6 concentrations at 0.5–1 h after an oral glucose tolerance test in obese individuals [5]. This acute decrease in plasma IL-6 is not due to hemodilution. In the present study, hematocrit did not change after ingestion of glucose solution and plasma IL-6 concentrations did not change appreciably after ingestion of a volume of water equivalent to the volume consumed during oral glucose intake. Furthermore, the small level of hemodilution during intravenous glucose administration as indicated by a 3% decrease in hematocrit, did not result in a decrease in circulating concentrations of IL-6.

The decrease in plasma IL-6 concentrations during oral glucose intake but not during intravenous glucose administration may be due, at least in part, to the 2.8-fold higher serum insulin concentrations following ingestion of glucose. Previous studies have reported anti-inflammatory activity of insulin [9], [11], [15], [16]. Nuclear transcription factor κB, the key cellular pro-inflammatory transcription factor, and the inflammatory marker monocyte chemotactic protein-1, were inhibited during a euglycemic, hyperinsulinemic clamp study in obese, nondiabetic subjects [9], [16]. In patients with type 2 diabetes, postprandial plasma IL-6 concentrations were lower when circulating insulin levels were approximately 2-fold higher during a test meal breakfast in those who were receiving pre-meal insulin therapy thrice daily compared with those who were receiving insulin only at bedtime [12]. This lower level of plasma IL-6 was correlated with the 64% lower post-meal hyperglycemia but not with the higher postprandial insulin levels suggesting that a decrease in post-meal hyperglycemia and not hyperinsulinemia directly, may be responsible for the lower plasma IL-6 levels [12]. In the present study, the difference in the response of plasma IL-6 concentrations between oral glucose intake and intravenous infusion of glucose cannot be readily attributed to change in plasma glucose levels as they were similar at all but one time point in the studies. Furthermore, the responses of plasma IL-6 and glucose concentrations during oral and intravenous glucose administration were unrelated. An insulin-mediated decrease in low-grade inflammation early after ingestion of nutrients that stimulate insulin secretion is also supported by a previous study that reported an acute 17% decrease in monocyte chemotactic protein-1 at 30 min-60 min after ingestion of meals rich in protein and fat in obese, nondiabetic subjects [17] when circulating insulin levels were high. Monocyte chemotactic protein-1 is a chemokine and a marker of inflammation [18] that contributes to macrophage infiltration into adipose tissue, adipose tissue inflammation, insulin resistance and hepatic steatosis in obesity [19], [20].

We cannot exclude the possibility that factors other than higher circulating insulin concentrations may contribute to the decrease in plasma IL-6 with oral intake but not during intravenous infusion of glucose. The response in plasma IL-6 was not associated with the response in serum insulin concentrations during the oral and intravenous intake of glucose. Oral but not intravenous intake of glucose and other nutrients stimulates the release of a number of gut peptides some of which might be capable of decreasing plasma IL-6 concentrations. Indeed, recent studies have suggested that pharmacologic agents that mimic or augment the action of the gut peptide glucagon like peptide – 1 have an anti-inflammatory effect [21], [22].

The physiological significance of an early postprandial decrease in plasma IL-6 is as yet uncertain. It is possible that this decrease represents a reduction in inflammation that allows insulin to more efficiently mediate the removal of high levels of glucose from the blood and perform its metabolic actions in adipose tissue and the liver. There is evidence that inflammation impairs insulin action [23]. In addition, people with Type 2 diabetes have impaired insulin secretion [24] and GLP-1 production [25] postprandially. Our findings also suggest that these abnormalities may exacerbate the inflammatory state and that interventions which increase insulin secretion and/or GLP-1 concentrations may have a beneficial effect.

This study has limitations. Participants were not randomized to the three treatment groups because our protocol required that blood glucose levels during the intravenous glucose treatment were matched to the corresponding levels during the oral glucose test. However, there was no evidence of appreciable carry-over from one intervention to the next. Also, we did not measure circulating incretin concentrations.

In conclusion, our data show that plasma IL-6 concentrations decrease after an oral glucose load but not during intravenous glucose administration suggesting that higher insulin levels and/or factors released from the gut may mediate a decrease in plasma IL-6 after ingestion of glucose.

## Supporting Information

Figure S1 **Plasma concentrations and incremental area under the curve for (A) glucose, (B) insulin, (C) nonesterified fatty acids, and (D) haematocrit following oral glucose (□) and intravenous (◊) glucose loads and oral water controls (•).** ^a,b^ Significantly different compared with intravenous at P = 0.03 and P<0.001 respectively. ^c^ Significantly different compared with oral at P = 0.004. ^d^ Significantly different compared with water at P<0.005.
(TIFF)

Figure S2 **Plasma IL-6 concentration and incremental area under the curve following oral glucose (□) and intravenous (◊) glucose loads and oral water controls (•).** *Significantly different from baseline at P<0.02 during oral glucose load. ^a^ Significantly different compared with intravenous at P = 0.005. ^b^ Significantly different compared with water at P = 0.02.
(TIFF)

Table S1 **Baseline characteristics of participants determined at the first visit.**
(DOCX)
