# Supplementary figures and images for: Oral but Not Intravenous Glucose Acutely Decreases Circulating Interleukin-6 Concentrations in Overweight Individuals
Source: PLoS One. 2013 Jun 12;8(6):e66395. doi: 10.1371/journal.pone.0066395 (PMC3680471; doi:10.1371/journal.pone.0066395)

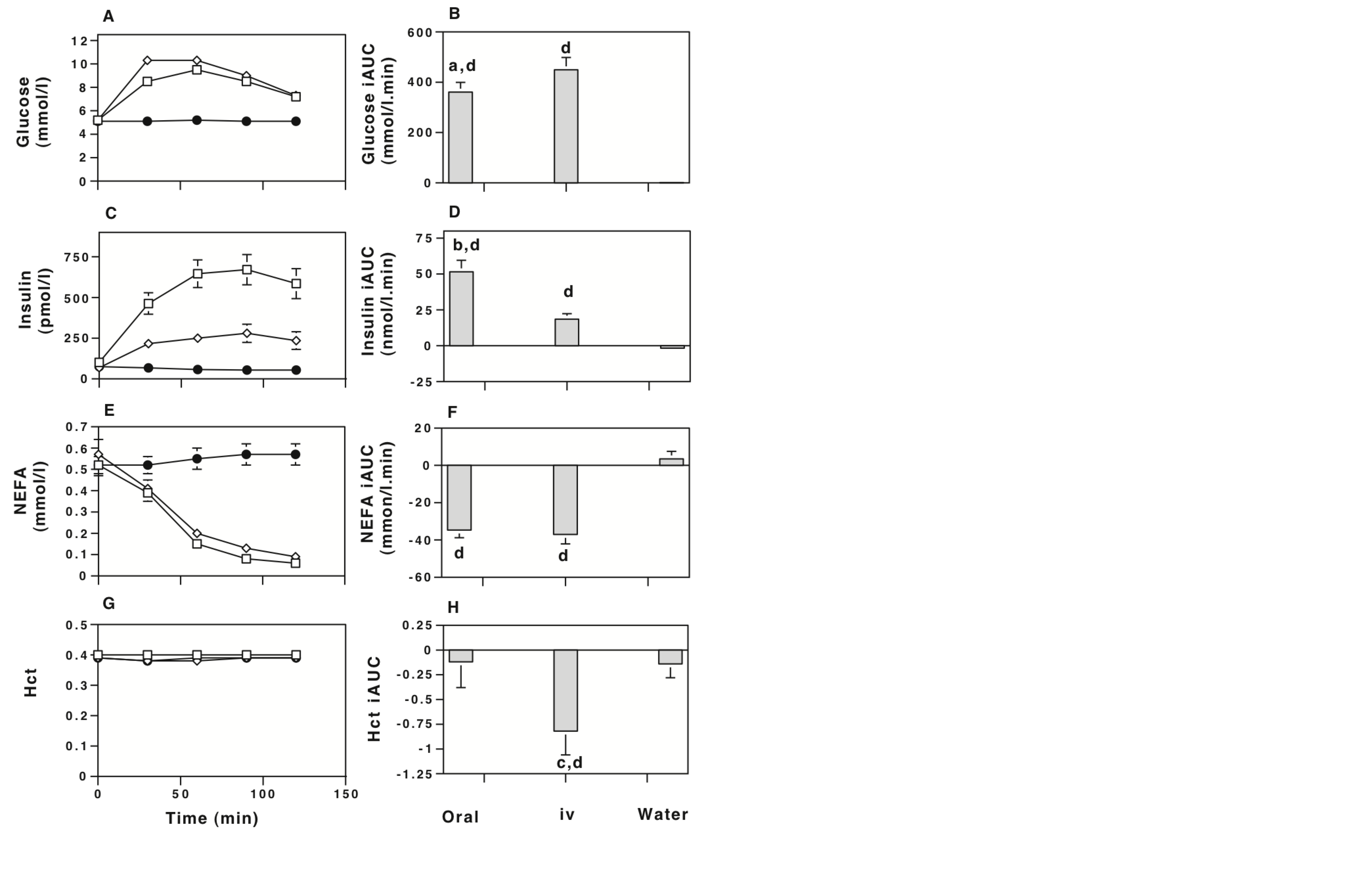

Supplement: Figure S1 — Plasma concentrations and incremental area under the curve for (A) glucose, (B) insulin, (C) nonesterified fatty acids, and (D) haematocrit following oral glucose (□) and intravenous (◊) glucose loads and oral water controls (•). a,b Significantly different compared with intravenous at P = 0.03 and P<0.001 respectively. c Significantly different compared with oral at P = 0.004. d Significantly different compared with water at P<0.005. (TIFF) [file pone.0066395.s001.tiff]

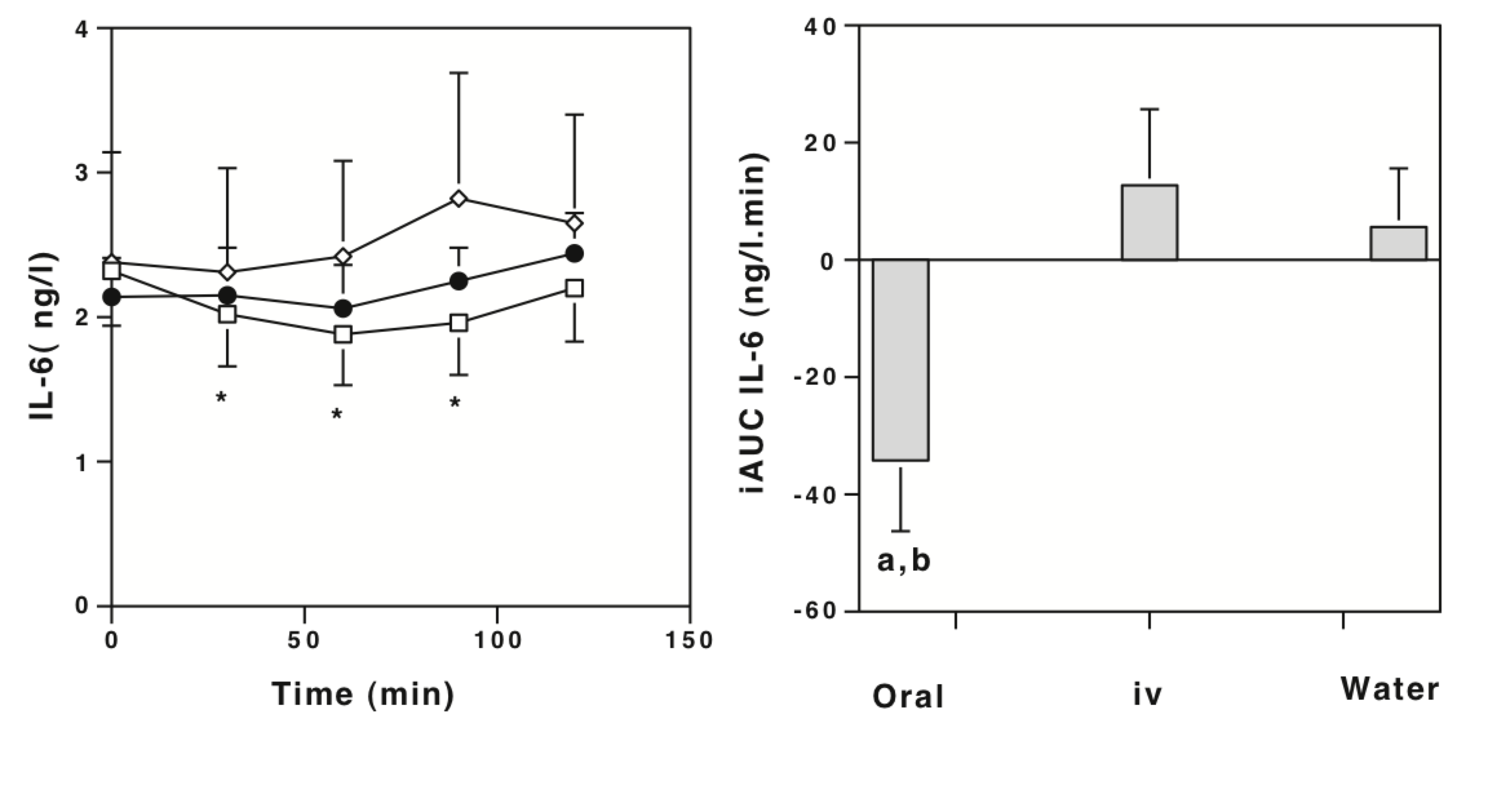

Supplement: Figure S2 — Plasma IL-6 concentration and incremental area under the curve following oral glucose (□) and intravenous (◊) glucose loads and oral water controls (•). *Significantly different from baseline at P<0.02 during oral glucose load. a Significantly different compared with intravenous at P = 0.005. b Significantly different compared with water at P = 0.02. (TIFF) [file pone.0066395.s002.tiff]
