# Supplementary material for: Oral but Not Intravenous Glucose Acutely Decreases Circulating Interleukin-6 Concentrations in Overweight Individuals
Source: PLoS One. 2013 Jun 12;8(6):e66395. doi: 10.1371/journal.pone.0066395 (PMC3680471; doi:10.1371/journal.pone.0066395)
Supplement: Table S1 — Baseline characteristics of participants determined at the first visit. (DOCX) [file pone.0066395.s003.docx]

**Table S1**. Baseline characteristics of participants determined at the first visit.

Age (y) 53±12.6

Sex (male/female) 3/12

BMI (kg/m^2^) 34.8±5.8

Systolic BP (mmHg) 125.6±10.3

Diastolic BP (mmHg) 77.3±7.3

Glucose (mmol/L) 5.1±0.6

Insulin (pmol/L) 100.87±83.8

IL-6 (ng/L) 2.3±1.4

NEFA (mmol/L) 0.51±0.2

Values are mean ± SD or numbers of participants. Abbreviations: BMI, body mass index; BP, blood pressure.
